# Cesium Inhibits Plant Growth through Jasmonate Signaling in *Arabidopsis thaliana*

**DOI:** 10.3390/ijms14034545

**Published:** 2013-02-25

**Authors:** Eri Adams, Parisa Abdollahi, Ryoung Shin

**Affiliations:** RIKEN Plant Science Center, 1-7-22 Suehiro-cho, Tsurumi-ku, Yokohama, Kanagawa 230-0045, Japan; E-Mails: eriadams@psc.riken.jp (E.A.); parisa@psc.riken.jp (P.A.)

**Keywords:** cesium, potassium, *Arabidopsis thaliana*, jasmonate, *HAK5*

## Abstract

It has been suggested that cesium is absorbed from the soil through potassium uptake machineries in plants; however, not much is known about perception mechanism and downstream response. Here, we report that the jasmonate pathway is required in plant response to cesium. Jasmonate biosynthesis mutant *aos* and jasmonate-insensitive mutant *coi1*-16 show clear resistance to root growth inhibition caused by cesium. However, the potassium and cesium contents in these mutants are comparable to wild-type plants, indicating that jasmonate biosynthesis and signaling are not involved in cesium uptake, but involved in cesium perception. Cesium induces expression of a high-affinity potassium transporter gene *HAK5* and reduces potassium content in the plant body, suggesting a competitive nature of potassium and cesium uptake in plants. It has also been found that cesium-induced *HAK5* expression is antagonized by exogenous application of methyl-jasmonate. Taken together, it has been indicated that cesium inhibits plant growth via induction of the jasmonate pathway and likely modifies potassium uptake machineries.

## 1. Introduction

Cesium (Cs^+^) is a Group I alkali metal, which exists naturally at very low concentrations in the soil. Cs^+^ has no known beneficial function in plants; however, it can, at high concentrations, cause toxicity, observed as growth inhibition (reviewed in [1]). Thus far, Cs^+^ uptake and response in plants have not been thoroughly studied. However, the accident at the Fukushima nuclear power plant following the disaster in 2011 in Japan highlighted the importance of understanding the mechanism of cesium uptake in plants to improve phytoremediation efficiency for radiocesium. Radiocesium, ^134^Cs and ^137^Cs, produced from anthropogenic sources are of environmental and health concerns, since they are rapidly incorporated into the food chain, emit β and γ radiation and have relatively long half-lives (reviewed in [1]). The potential of plants to decontaminate radiocesium from soil was also focused after the accident in Chernobyl [2,3]. Earlier studies have suggested that Cs^+^ may be absorbed by plants in a competitive fashion with potassium (K^+^), which belongs to the same alkali metal group and shares similar chemical properties with Cs^+^. The first report that plants accumulate Cs^+^ through the same uptake mechanism as K^+^ was published as early as 1941 [4], and many other studies reinforced this view (reviewed in [1]). A theoretical modeling predicted that voltage-insensitive cation channels (VICCs) predominantly mediate Cs^+^ uptake under the K^+^ sufficient conditions and K^+^ uptake permeases (KUPs) significantly contribute under the K^+^ deficient conditions [1]. In *Arabidopsis thaliana*, some members of cyclic nucleotide gated channels (CNGCs), which belong to VICCs, such as CNGC1, CNGC2, CNGC4 and CNGC10, were predicted to form K^+^ channels and, thus, suggested as being involved in Cs^+^ uptake, also [5–8].

The first K^+^ channel identified in plants is KAT1 from *Arabidopsis*, which functions at high K^+^ concentrations as a voltage-dependent, low-affinity inward rectifying channel [9]. The major K^+^ channel functioning in the roots of *Arabidopsis* under the K^+^ sufficient conditions is assumed to be AKT1 [10]. However, the contribution of these channels to Cs^+^ uptake is small or negligible [9,11]. In response to K^+^ deficiency, expression of high-affinity K^+^ transporters, members of the KUP family, are induced, such as high affinity K^+^ transporter5 (*HAK5*) and *KUP3*[12,13]. HAK5 has been reported to be involved in Cs^+^ uptake *in planta*, consequently resulting in higher Cs^+^ accumulation under the K^+^ deficient conditions [12]. Another K^+^ transporter, KUP9, from *Arabidopsis* was also shown to transport Cs^+^ when expressed in a K^+^ transport-deficient mutant of *Escherichia coli*[14].

There have been a few reports pointing out interaction between K^+^ deficiency response and phytohormone signaling. Expression of ethylene biosynthesis genes and ethylene production are reported to be induced in response to K^+^ deficiency and, subsequently, activate reactive oxygen species production [15]. Expression of jasmonate (JA) biosynthesis genes is also induced in response to K^+^ deficiency and confers tolerance to insects [16–18]. Cytokinins, by contrast, decrease under the K^+^ deficient conditions and allow downstream response for adaptation to take place [19]. Auxin-related mutants were found to be impaired in K^+^ deficiency-induced lateral root growth [20]. If Cs^+^ were to cause similar effects as K^+^ deficiency in plants, it is possible that Cs^+^ response is regulated through phytohormone pathways.

In this study, JA biosynthesis and signaling were demonstrated to be required in plant response to Cs^+^. JAs are a class of phytohormone that are involved in various physiological processes, such as growth inhibition, fertility, senescence and defense against biotic and abiotic stresses [21]. JAs are biosynthesized from linolenic acid through a series of steps, including allene oxide synthase (AOS) [22], and recognized by a receptor complex which is composed of an E3 ubiquitin ligase, SCF (SKP1, CDC53p/CUL1 F-box protein) complex. An active form of JAs, the jasmonyl-isoleucine conjugate (JA–Ile), directly binds to the F-box protein in the receptor complex, CORONATINE INSENSITIVE1 (COI1), and SCF^COI1^ targets the negative regulators for proteolysis to activate downstream JA signaling in *Arabidopsis*[23–27]. Therefore, mutations in *COI1* can render the plants insensitive to JAs [28,29].

Here, we investigated the relationship between JA biosynthesis/signaling and Cs^+^ response in *Arabidopsis* in order to understand the fundamental mechanism of plant response to Cs^+^. Our studies revealed the induction of JA biosynthesis/signaling and consequent growth inhibition in response to Cs^+^. However, JA signaling was not apparently involved in Cs^+^ uptake under the K^+^ sufficient conditions. Meanwhile, induction of *HAK5* was demonstrated in response to Cs^+^, suggesting the involvement of K^+^ uptake machineries in Cs^+^ uptake. The possibility of JA signaling casting an antagonistic effect on *HAK5* expression was also implied. In this report, plant response to Cs^+^ is dissected.

## 2. Results

### 2.1. JA-Related Mutants Are Less Responsive to Cs^+^-Induced Growth Inhibition

**Figure 1:**
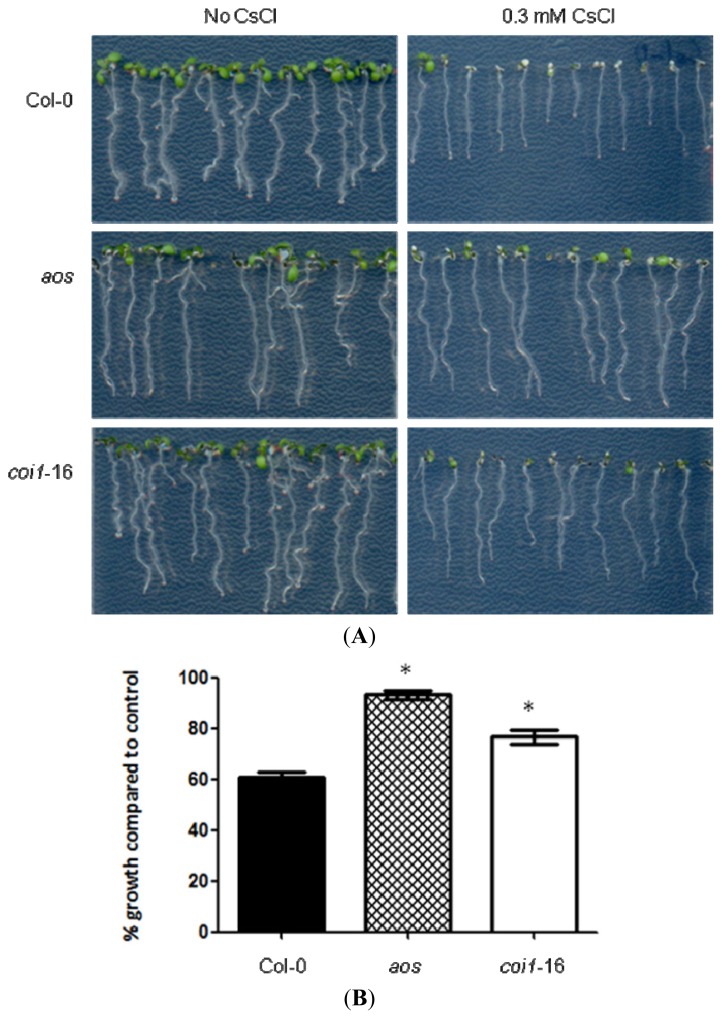
(**A**) Phenotype of Col-0 (wild type), *aos* and *coi1*-16 grown on 0.5 mM KCl with or without 0.3 mM CsCl for seven days. (**B**) Primary root % growth on 0.3 mM CsCl compared to the control condition. Primary root lengths of the seedlings treated with or without cesium were measured, and the % growth was calculated for Col-0 (black bar), *aos* (crisscross bar) and *coi1*-16 (white bar). Error bars indicate standard error (*n* ≥ 20). Statistically significant differences (*p <* 0.001) compared to Col-0 are indicated as asterisks.

In order to understand the mechanism of Cs^+^ response in plants, phytohormone-related mutants in *Arabidopsis thaliana* were tested on 0.5 mM KCl with or without 0.3 mM CsCl. This condition was chosen as a “stringent condition” for monitoring strong Cs^+^ resistance phenotype. In this condition, growth of Col-0 plants (wild-type) was severely retarded by the addition of CsCl (Figure 1A). However, a JA biosynthesis mutant *aos*[30,31] and a JA-insensitive mutant *coi1*-16 [29] showed significantly less response to Cs^+^-induced root growth retardation (*p <* 0.001, Figure 1). A mutant, which produces less bioactive JA-Ile and shows mild JA-insensitive phenotype, *jasmonate resistant1* (*jar1*-1) [32], also showed less response to Cs^+^ in terms of root growth inhibition (*p <* 0.001, Table S1). Among them, the stronger phenotype of *aos* corresponds with the fact that the *aos* mutant is a knockout line [30]. The *coi1*-16 and *jar1*-1 mutants are known to be “leaky” [29,33], and this might be why their resistance to Cs^+^ was also compromised. Also, other phytohormone-related mutants were found to be less responsive to Cs^+^, such as an auxin-insensitive mutant, *auxin resistant1* (*aux1*-7) [34] (*p <* 0.001), and, to a lesser extent (*p <* 0.01), a *trans*-zeatin-type cytokinin biosynthesis quadruple mutant, *isopentenyltransferase1*,*3*,*5*,*7* (*ipt1,3,5,7*) [35] and a cytokinin-insensitive double mutant *histidine kinase2;3* (*ahk2;3*) [36] (Table S1). An ethylene-insensitive mutant, *ethylene insensitive2,* (*ein2*-1) [37], but not *ethylene response sensor1* (*ers1*-1) [38], was marginally less responsive to Cs^+^ (*p <* 0.05, Table S1). A GA-related DELLA double mutant, *repressor of ga;gibberellic acid insensitive* (*rga*-24;*gai*-t6) [39], showed increased growth % in response to Cs^+^ compared to its wild-type, L*er* (*p <* 0.001, Table S1). This could be due to the absence of growth inhibitor proteins in the mutant [39], enabling the resistance to Cs^+^-induced growth inhibition, but further analysis is required for confirmation. The relationship between Cs^+^ response and JA signaling was further investigated.

### 2.2. Resistance of JA-Related Mutants to Cs^+^ Was Not Due to Less Uptake of Cs^+^

**Figure 2:**
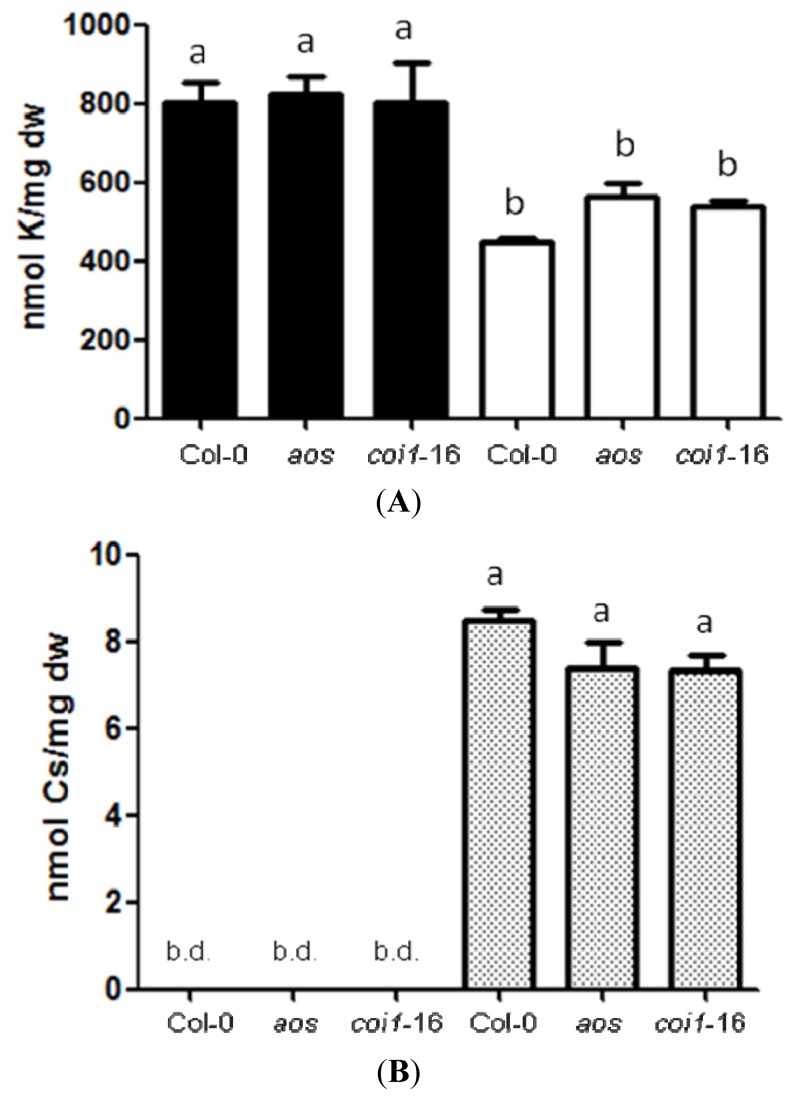
(**A**) K^+^ contents in Col-0, *aos* and *coi1*-16 grown on 1.75 mM KCl with (white bars) or without (black bars) 0.3 mM CsCl for seven days. (**B**) Cs^+^ contents in Col-0, *aos* and *coi1*-16 grown on 1.75 mM KCl with (dotted bars) or without 0.3 mM CsCl for seven days. Error bars indicate standard error for four biological replicates. Each sample contained more than 15 seedlings. b.d.: below the detection limit. Alphabetical letters show statistical differences (*p <* 0.05).

JA-related mutants were found to be less responsive to Cs^+^-induced growth inhibition compared to wild-type. One explanation could be that JA-related mutants might be impaired in Cs^+^ uptake. To test this possibility, K^+^ and Cs^+^ contents were measured in wild-type, *aos* and *coi1*-16 treated with or without 0.3 mM CsCl under the K^+^ sufficient condition (1.75 mM KCl) for seven days. Reduced K^+^ contents were observed upon Cs^+^ treatment (*p <* 0.05, Figure 2A). However, there was no statistically significant difference observed between wild-type and mutants for both K^+^ and Cs^+^ contents, regardless of Cs^+^ treatment (Figure 2). This suggests that the JA pathway is not involved in the uptake of Cs^+^ or K^+^ in this condition. More K^+^ and Cs^+^ were accumulated per mg dry weight at the later stage of growth (day 12), but the pattern was maintained (Figure S1).

### 2.3. Cs^+^ Induces JA Signaling as well as *HAK5* Expression

**Figure 3:**
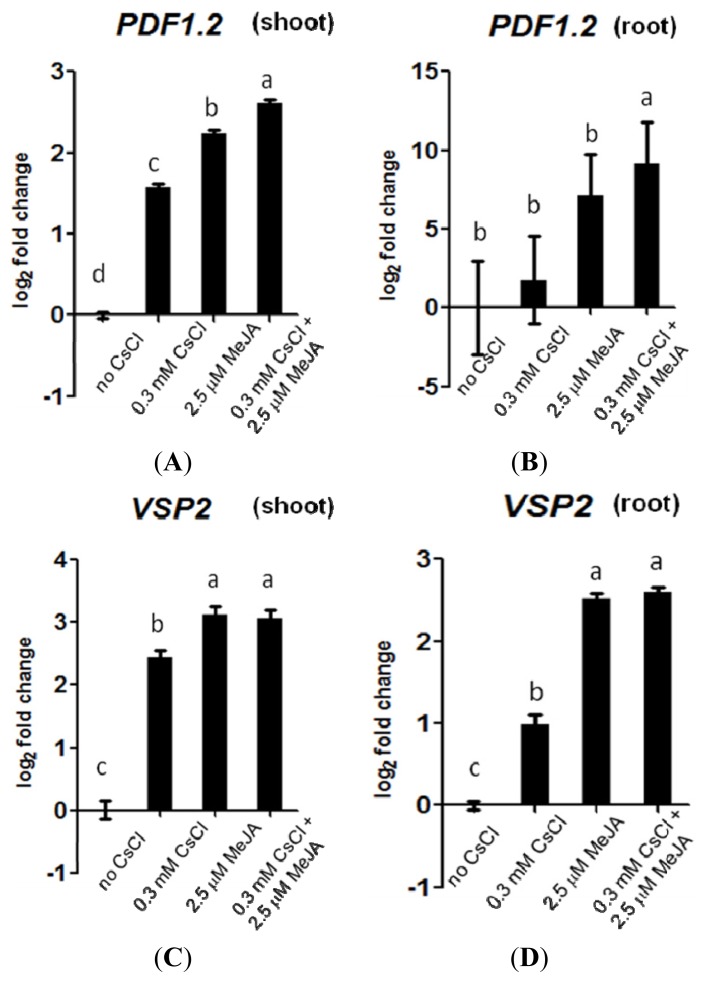
(**A**) Gene expression of *PDF1.2* in Col-0 shoots and (**B**) roots, and (**C**) gene expression of *VSP2* in Col-0 shoots and (**D**) roots, grown on 1.75 mM KCl with or without 0.3 mM CsCl and 2.5 mM methyl jasmonate (MeJA) for 11 days. Values are log_2_ ratios relative to the control. Error bars indicate standard error for three technical replicates. Each sample contained more than 15 seedlings, and the experiment was repeated three times, one of which is presented. Alphabetical letters show statistical differences (*p <* 0.05).

To further analyze the interplay between Cs^+^ perception and JA signaling, expression of the marker genes for JA signaling, *PDF1.2* and *VSP2*[40–42], was investigated in response to Cs^+^. Both *PDF1.2* and *VSP2* levels were increased upon Cs^+^ treatment under the K^+^ sufficient condition, especially in the shoots (*p <* 0.05, Figure 3), suggesting the induction of the general JA pathway by Cs^+^. This induction was absent in the *aos* mutant (Figure S2). There was no additive effect of Cs^+^ on methyl jasmonate (MeJA)-induced *VSP2* expression (Figure 3).

**Figure 4:**
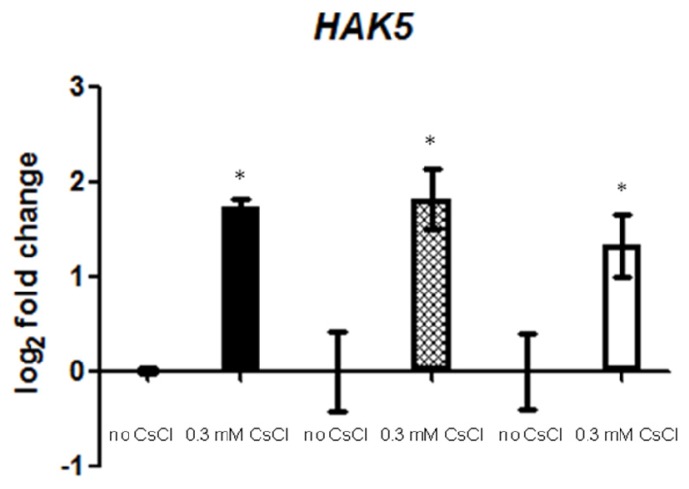
Gene expression of *HAK5* in Col-0 (black bar), *aos* (crisscross bar) and *coi1*-16 (white bar) grown on 1.75 mM KCl with or without 0.3 mM CsCl for seven days. Values are log_2_ ratios relative to the controls. Error bars indicate standard error for three technical replicates. Each sample contained more than 15 seedlings, and the experiment was repeated three times, one of which is presented. Statistically significant differences (*p <* 0.05) compared to each non-treated sample are indicated as asterisks.

It has been reported that high-affinity K^+^ transporter, HAK5, is not only involved in K^+^ uptake, but also in Cs^+^ uptake [12]. The expression of *HAK5* is sharply induced in response to K^+^ deficiency [12], and there are some indications that Cs^+^ may cause K^+^ deficiency in plants. Therefore, we analyzed the transcript levels of *HAK5* in response to Cs^+^ under the K^+^ sufficient condition. Expression of *HAK5* was, indeed, induced in response to Cs^+^, and this induction also occurred in JA-related mutants, *aos* and *coi1*-16 (*p <* 0.05, Figure 4).

### 2.4. JA Antagonizes Cs^+^-Induced *HAK5* Expression

**Figure 5:**
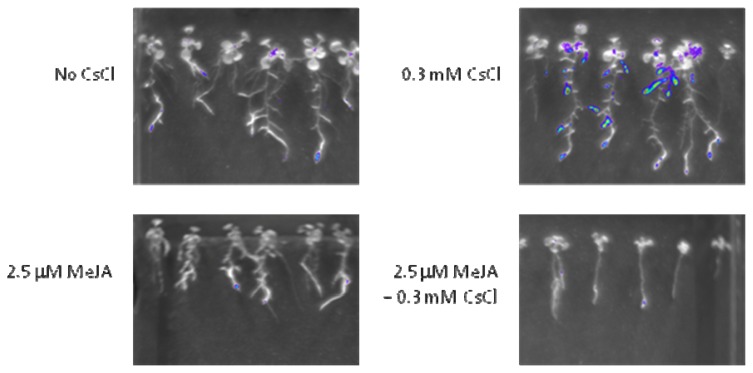
Chemiluminescence imaging of *HAK5promoter::LUC* plants grown on 1.75 mM KCl with or without 0.3 mM CsCl and 2.5 mM MeJA for 11 days. Pseudo color represents the intensity of chemiluminescence.

Since *HAK5* was found to be induced in response to Cs^+^, spatial localization of *HAK5* was investigated. Transgenic plants carrying the *HAK5* promoter fused to the luciferase reporter construct (*HAK5promoter::LUC*) were used, and their activities are known to be induced in K^+^ deficiency [15]. In this study, low levels of luciferase activity were visible at root tips of untreated plants under the K^+^ sufficient condition (Figure 5). Upon Cs^+^ treatment, strong induction of luciferase activity was observed in the roots, and this induction was abolished by the addition of MeJA (Figure 5). The chemiluminescence intensity of these images and quantification of *HAK5* transcripts in wild-type roots treated with Cs^+^ and MeJA are shown in Table S2 and Figure S3, respectively. Chemiluminescence images of *HAK5promoter::LUC* plants grown on 0.5 mM KCl with or without Cs^+^ are also presented in Figure S4.

## 3. Discussion

Cs^+^ has no known nutritional or other function in plants. Thus far, scarcely any knowledge has been gained on how plants absorb Cs^+^ from the soil, how it is transported inside the plant body, and what is the plant response. However, an urgent need for improved phytoremediation methods for radioactive Cs^+^ has urged scientists to better understand the mechanism of Cs^+^ uptake, transport and perception in plants. In *Arabidopsis*, a possible mechanism for tolerance to heavy metals, such as arsenic, mercury and cadmium, has been reported [43,44], but whether the same tolerance mechanism applies for Cs^+^ is not yet known. A T-DNA insertion mutant which is impaired in Cs^+^ uptake has been isolated; however, the identity of the gene disturbed is not revealed [45].

The similarity of the nature between Cs^+^ and K^+^ and the involvement of phytohormone pathways in K^+^ deficiency response provoked us to investigate the relationship between phytohormone pathways and Cs^+^ response. For this purpose, various phytohormone-related mutants in *Arabidopsis thaliana* were analyzed for their response to Cs^+^. It has been reported that K^+^ deficiency response is observed under 100 μM K^+^ concentrations in *Arabidopsis*[12]. Therefore, we used the 0.5 mM KCl condition, which is well above the threshold for “K^+^ deficient conditions”, but lower than what is generally taken as “K^+^ sufficient conditions” (1.75 mM KCl) [12], to create a “stringent condition” in selecting strong phenotype mutants for Cs^+^. Of those mutants tested, JA-related mutants, *aos*, *coi1*-16 and *jar1*-1, showed strong resistance to Cs^+^-induced growth inhibition. Our data suggest that the JA pathway, and possibly some other phytohormone pathways, is involved in plant response to Cs^+^. This is the first report, as far as we are aware, to demonstrate the interaction between Cs^+^ response and the JA pathway in plants. Some phytohormone pathways, including ethylene, auxin, cytokinin and JA, have been reported to be involved in response to K^+^ deficiency [15–20]; therefore, in the future, it will be interesting to investigate the relationship of the Cs^+^-induced JA pathway with K^+^ deficiency.

Although *aos* and *coi1*-16 were less responsive to Cs^+^, this was not because they accumulated less Cs^+^. Cs^+^ contents, as well as K^+^ contents, were comparable to those of wild-type plants, suggesting that the JA pathway is involved, at least partly, in the perception of Cs^+^, not in uptake. Classic downstream marker genes of the JA pathway, *PDF1.2* and *VSP2*, represent two distinct branches of the signaling pathway [40–42]. Under the K^+^ sufficient condition, both of these marker genes were found to be increased in response to Cs^+^, especially in the aerial parts. This induction was not observed in the *aos* mutant, indicating that Cs^+^ induces JA biosynthesis and downstream signaling.

It was also interesting to observe that K^+^ contents were reduced upon Cs^+^ treatment under the K^+^ sufficient condition. The competitive nature between Cs^+^ and K^+^ accumulation in *Arabidopsis* was demonstrated in a previous report [46]. Although Cs^+^ has been used as a classic pharmacological inhibitor of K^+^ channels [47], our findings might also indicate that Cs^+^ could have been absorbed through the K^+^ uptake mechanism in a competitive manner. There are a few reports that suggest the possibility of K^+^ transporters/channels absorbing Cs^+^ in plants, including a low-affinity inward-rectifying channel in *Arabidopsis*, KAT1 [9], a high-affinity K^+^ transporter, HAK5 [12,48], and another K^+^ transporter, KUP9 [14]. In this study, we indicated that the transcript levels of *HAK5* were increased in response to Cs^+^ under the K^+^ sufficient condition. The reporter construct study using *HAK5promoter::LUC* showed that its promoter activity was particularly notable in the actively growing part of the roots, and this pattern was similar to that of the K^+^-starved plants [15]. These findings, together with the results from the K^+^/Cs^+^ content study, agree with the hypothesis that Cs^+^ is absorbed through K^+^ uptake machinery in a competitive fashion.

JA treatment, on the other hand, was found to abolish Cs^+^-induced *HAK5* expression in the reporter construct study, suggesting an antagonistic effect of JA on *HAK5* expression. Since Cs^+^ seemingly induces both JA biosynthesis/signaling and *HAK5* expression, this antagonism may function as a negative feedback mechanism to fine-tune Cs^+^ response in plants.

**Figure 6:**
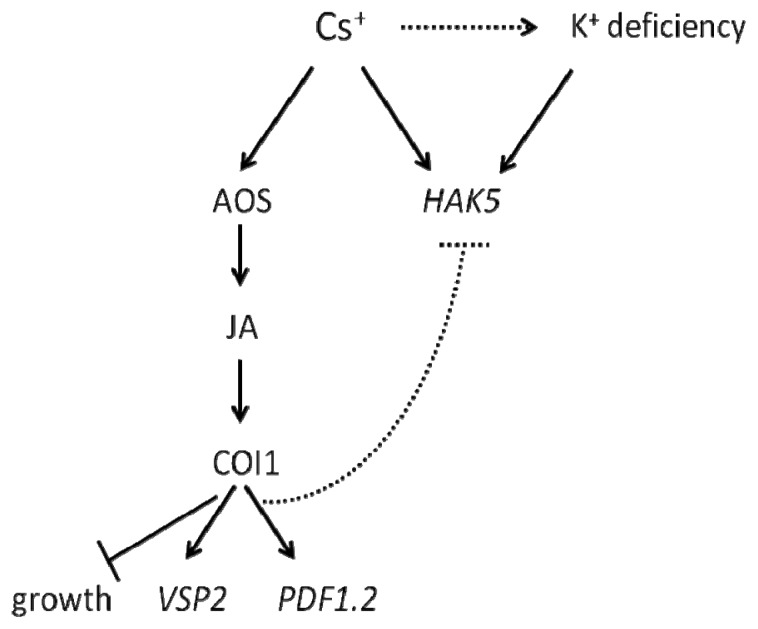
Model pathway. Cs^+^ induces the JA pathway and inhibits plant growth. Cs^+^ also induces *HAK5* expression. Whether this expression is due to Cs^+^-induced K^+^ deficiency or the direct effect of Cs^+^ is not yet clear. An antagonistic effect of the JA pathway on *HAK5* expression is also suggested.

A summary of our findings on Cs^+^-induced growth inhibition and gene expression is shown as a model pathway in Figure 6. Cs^+^ induces JA biosynthesis and the downstream signaling pathway in *Arabidopsis*. This is probably the major pathway for Cs^+^-induced growth inhibition of roots, since a mutant incapable of producing JAs hardly shows any reduction in root length in response to Cs^+^. In parallel, Cs^+^ induces *HAK5*, a reporter gene for K^+^ deficiency [49]. Further studies are required to elucidate whether Cs^+^ treatment causes K^+^ deficiency to induce *HAK5* expression in plants. It will be interesting to test plant response to Cs^+^ under various K^+^ conditions, since plants are expected to exhibit different K^+^ uptake mechanisms, according to K^+^ availability. It was also indicated that the JA pathway might antagonize *HAK5* expression. The mechanism in which this antagonism occurs awaits elaboration; however, it is possible that one of APETALA2/Ethylene Response Factor (AP2/ERF) transcription factors induced by the JA signaling pathway binds to the GCC-box of the *HAK5* promoter to regulate its expression. *HAK5* has been reported to be regulated by an ERF/AP2 transcription factor, RAP2.11, in response to K^+^ deficiency [50]. Meanwhile, JA-inducible AP2/ERFs are known to regulate expression of a series of GCC-box-containing genes, such as *PDF1.2*, through direct binding to the GCC-boxes of the promoter region [51–53]. Recently, involvement of cytokinin in K^+^ deficiency response and *HAK5* expression has been reported [19]. It will be also interesting to further investigate the involvement of biosynthesis and the signaling of cytokinin and other phytohormones in Cs^+^ response in plants.

Here, we presented the first report that demonstrated the interplay between Cs^+^ response and JA signaling in plants. It was also highlighted that Cs^+^ uptake, Cs^+^-induced growth inhibition and gene expression were apparently regulated through different mechanisms. Elucidation of the fine balance between K^+^ and Cs^+^ to regulate plant response and growth is awaited.

## 4. Experimental Section

### 4.1. Plant Material and Growth Conditions

The *Arabidopsis thaliana* (L.) Heynh. accession Col-0 and L*er* were used as wild-types. *HAK5promoter::LUC* was developed in the lab [15]. *aos*[30,31], *ein2*-1 [37] and *jar1*-1 [32] are NASC stocks (Nottingham Arabidopsis Stock Centre, Loughborough, UK). *coi1*-16 [29], *ipt1,3,5,7*[35], *ahk2;3*[36], *ers1*-1 [38], *aux1*-7 [34] and *rga*-24;*gai*-t6 [39] were gifts from Dr. Turner, Dr. Kakimoto, Dr. Wen, Dr. Schachtman and Dr. Harberd. Seeds were surface-sterilized with 70% (*v/v*) ethanol and 0.05% (*v/v*) Triton X-100 and sown on media, indicated below. For the growth assay, media contained 0.5 mM KCl, 2 mM Ca(NO_3_)_2_, 0.5 mM phosphoric acid, 0.75 mM MgSO_4_, 50 μM H_3_BO_3_, 10 μM MnCl, 2 μM ZnSO_4_, 1.5 μM CuSO_4_, 0.075 μM NH_4_Mo_7_O_24_ and 74 μM Fe-EDTA, pH 5.8, with Ca(OH)_2_, 1% (*w/v*) sucrose and 0.5% (*w/v*) SeaKem agarose (Cambrex, Rockland, ME, USA) supplemented with or without 0.3 mM CsCl and for the rest of the study, media contained 1.75 mM KCl, 2 mM Ca(NO_3_)_2_, 0.5 mM phosphoric acid, 0.75 mM MgSO_4_, 50 μM H_3_BO_3_, 10 μM MnCl, 2 μM ZnSO_4_, 1.5 μM CuSO_4_, 0.075 μM NH_4_Mo_7_O_24_ and 74 μM Fe-EDTA, pH 5.8, with Ca(OH)_2_, 1% (*w/v*) sucrose and 0.5% (*w/v*) SeaKem agarose (Cambrex) supplemented with or without 0.3 mM CsCl or 2.5 μM MeJA. After stratifying for 3 to 4 days at 4 °C, plants were placed in a vertical orientation in a growth cabinet at 22 °C in a 16 h light/8 h dark photocycle with a light intensity of 70–90 μmol/m^2^/sec.

### 4.2. Root Growth Assay

Seedlings grown for 7 days were removed from media, and the root lengths were measured directly on a ruler wetted with 70% (*v/v*) glycerol. The relative % of root growth was calculated according to the formula, *y*/*x* × 100, where *x* was the average of root lengths of untreated seedlings and *y* was the root lengths of each Cs^+^-treated seedling and was averaged. One-way ANOVA with Dunnett’s multiple comparison posttest (*p <* 0.05) was performed using Prism (GraphPad Software, La Jolla, CA, USA) to determine the statistical significance.

### 4.3. Element Analysis

Seedlings grown for 7 days were washed in Milli-Q water, dried on a piece of paper towel, placed in a paper envelope and dried in an oven at 65 °C for 3–4 days. Approximately 2 mg of dried samples were extracted in 1 mL of 60% (*v/v*) HNO_3_ at 125 °C for 1 h, followed by 1 mL of 30% (*v/v*) H_2_O_2_ and diluted with Milli-Q water to get a total volume of 10 mL. For K^+^ analysis, samples were further diluted 10 times with 6% (*v/v*) HNO_3_. For Cs^+^ analysis, 0.1% (*w/v*) KCl was added to each sample and standard solution to prevent ionization of Cs^+^, according to the manufacturer’s instructions (PerkinElmer, Waltham, MA, USA). K^+^ and Cs^+^ contents were measured on a flame atomic absorption spectrometer AAnalyst 200 (PerkinElmer). Concentrations were calculated against each standard curve, and one-way ANOVA with Bonferroni′s multiple comparison posttest (*p <* 0.05) was performed using Prism to determine the statistical significance.

### 4.4. qRT-PCR Analysis

Seedlings grown for 7 days (for *HAK5* expression) or 11 days (for *VSP2* and *PDF1.2* expression) were flash-frozen in liquid N_2_ and ground using a mixer mill. Total RNA was extracted, treated with DNaseI (Invitrogen, Carlsbad, CA, USA) and synthesized into cDNA using SuperScript III (Invitrogen). Quantitative real-time reverse transcription-PCR (qRT-PCR) was performed using THUNDERBIRD SYBR qPCR mix (TOYOBO, Osaka, Japan) and a Mx3000P qPCR system (Agilent Technologies, Santa Clara, CA, USA). The amplification conditions were 95 °C for 15 s and 60 °C for 30 s. The cycle was repeated 40 times, preceded by 95 °C for 1 min and followed by a dissociation program to create melting curves. Three technical replicates for each sample were run. The β-tubulin gene (*TUB2*) was used as a reference gene. The primers used were as follows: HAK5 forward 5’-CGAGACGGACAAAGAAGAGGAACC and reverse 5’-CACGACCCTTCCCGACCTAATCT [49]; VSP2 forward 5’-CCTAAAGAACGACACCGTCA and reverse 5’-TCGGTCTTCTCTGTTCCGTA [54]; PDF1.2 forward 5’-TTGCTGCTTTCGACGCA and reverse TGTCCCACTTGGCTTCTCG [51]; and TUB2 forward 5’-GCCAATCCGGTGCTGGTAACA and reverse 5’-CATACCAGATCCAGTTCCTCCTCCC [49]. One-way ANOVA with Bonferroni’s multiple comparison posttest (*p <* 0.05) was performed using Prism to determine the statistical significance.

### 4.5. Luciferase Imaging

*HAK5promoter::LUC* grown for 11 days were sprayed with luciferin (Duchefa Biochemie, Haarlem, The Netherlands) and kept in the dark for a few minutes prior to imaging. NightSHADE LB 985 (Berthold, Bad Wildbad, Germany) was used to image luciferase chemiluminescence.

## 5. Conclusions

In this study, we have determined that JA signaling is involved in Cs^+^ perception in terms of root growth inhibition, but not in uptake of K^+^ and Cs^+^. This growth inhibition by Cs^+^ occurs presumably through biosynthesis of JAs and consequent stimulation of the downstream JA signaling pathway. In parallel, Cs^+^ induces expression of a high-affinity K^+^ transporter gene, *HAK5*, under the K^+^ sufficient condition in a JA signaling-independent manner. However, exogenous application of MeJA inhibits Cs^+^-induced expression of *HAK5*, suggesting a complicated “fine-tuning” regulation of Cs^+^ response in plants.

## Supplementary Information
