# Supplementary material for: Cesium Inhibits Plant Growth through Jasmonate Signaling in Arabidopsis thaliana
Source: Int J Mol Sci. 2013 Feb 25;14(3):4545–59. doi: 10.3390/ijms14034545 (PMC3634425; doi:10.3390/ijms14034545)
Supplement: Supplementary file 1 [file ijms-14-04545-s001.docx]

**Supporting Information**

**Table S1.** Primary root % growth on 0.3 mM CsCl compared to the control condition. Primary root lengths of the seedlings grown on 0.5 mM KCl with or without cesium for
7 days were measured and the % growth was calculated for Col-0, *ipt1,3,5,7*, *ahk2;3,* *ein2*-1, ers*1*-1, *aux1*-7, *jar1*-1, L*er* (wild type for *rga*24;*gai*t6) and *rga*24;*gai*t6. Standard error
(*n* ≥ 18) and *P*-values compared to each wild type are indicated.

|  | **Growth %** | **SE** | ***P*-value** |
| --- | --- | --- | --- |
| Col-0 | 61.07 | 1.65 |  |
| *ipt1,3,5,7* | 72.41 | 2.67 | <0.01 |
| *ahk2;3* | 72.79 | 3.88 | <0.01 |
| Col-0 | 65.61 | 1.21 |  |
| *ers1*-1 | 73.33 | 2.51 | >0.05 |
| *ein2*-1 | 73.87 | 3.81 | <0.05 |
| *aux1*-7 | 80.30 | 3.56 | <0.001 |
| *jar1*-1 | 81.42 | 2.38 | <0.001 |
| L*er* | 87.98 | 1.54 |  |
| *rga*24*;gait*6 | 110.68 | 1.93 | <0.001 |

**Figure S1.** (**A**) K^+^ contents in Col-0, *aos* and *coi1*-16 grown on 1.75 mM KCl with (white bars) or without (black bars) 0.3 mM CsCl for 12 days; (**B**) Cs^+^ contents in Col-0, *aos* and *coi1*-16 grown on 1.75 mM KCl with (dotted bars) or without 0.3 mM CsCl for 12 days. Error bars indicate standard error for three biological replicates. Each sample contained more than 15 seedlings. b.d. indicates below the detection limit.

| 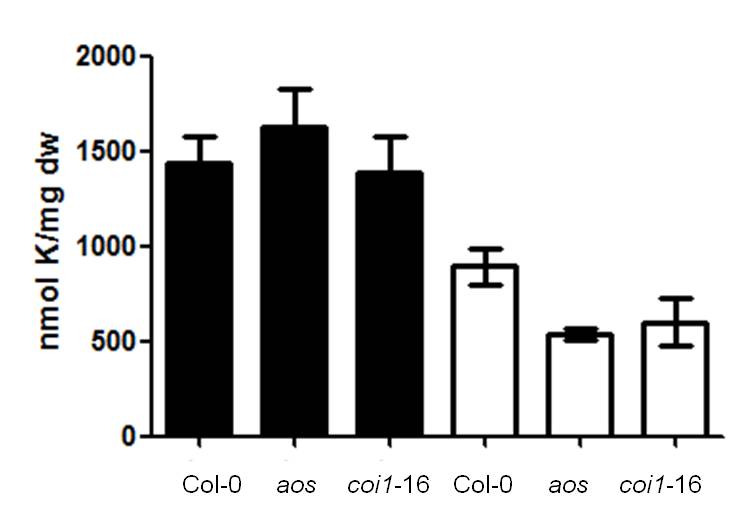 | 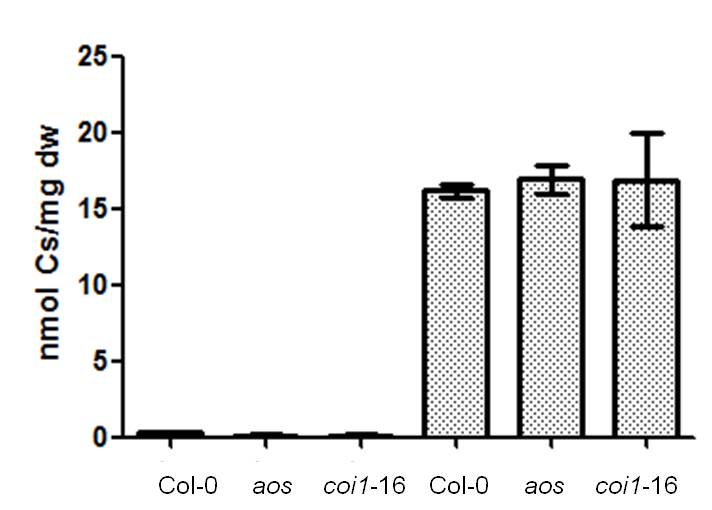 |
| --- | --- |
| (**A**) | (**B**) |

**Figure S2.** Gene expression of *PDF1.2* and *VSP2* in *aos*, grown on 1.75 mM KCl with or without 0.3 mM CsCl for 7 days. Values are log_2_ ratios relative to the control. Error bars indicate standard error for three technical replicates. Each sample contained more than
15 seedlings.


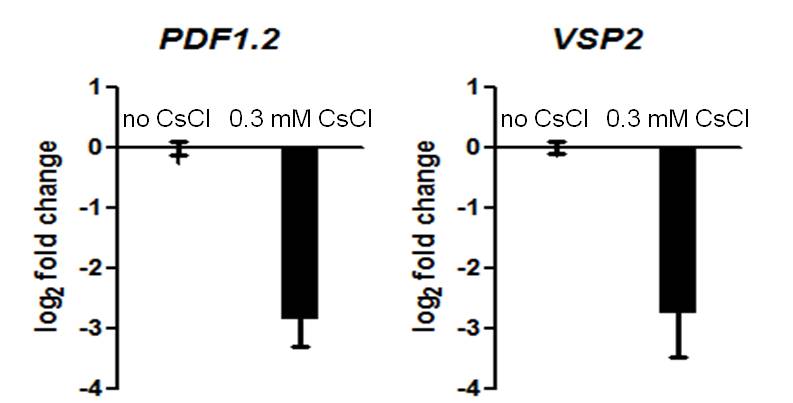


**Table S2.** Chemiluminescence intensity of *HAK5promoter::LUC* plants grown on 1.75 mM KCl with or without 0.3 mM CsCl and 2.5 μM MeJA for 11 days (cf. Figure 5 for imaging). Values are counts per second (cps) obtained using IndiGo software (Berthold).

| **Treatment** | **Overall cps** |
| --- | --- |
| no CsCl | 519 |
| 0.3 mM CsCl | 6767 |
| 2.5 μM MeJA | 38 |
| 0.3 mM CsCl + 2.5 μM MeJA | 20 |

**Figure S3.** Gene expression of *HAK5* in Col-0 roots, grown on 1.75 mM KCl with or without 0.3 mM CsCl and 2.5 μM MeJA for 11 days. Values are log_2_ ratios relative to the control. Error bars indicate standard error for three technical replicates. Each sample contained more than 15 seedlings.


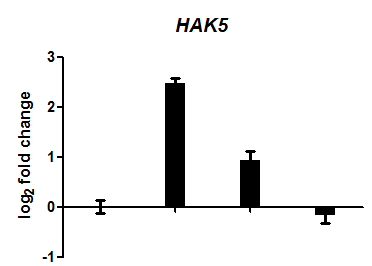


**no CsCl**

**0.3 mM CsCl**

**0.3 mM CsCl + 2.5 μM MeJA**

**2.5 μM MeJA**

**Figure S4.** Chemiluminescence imaging of *HAK5promoter::LUC* plants grown on
0.5 mM KCl with or without 0.3 mM CsCl for 7 days. Pseudo colour represents the intensity of chemiluminescence. (**A**) 0.5 mM KCl; (**B**) 0.5 mM KCl + 0.3 mM CsCl.


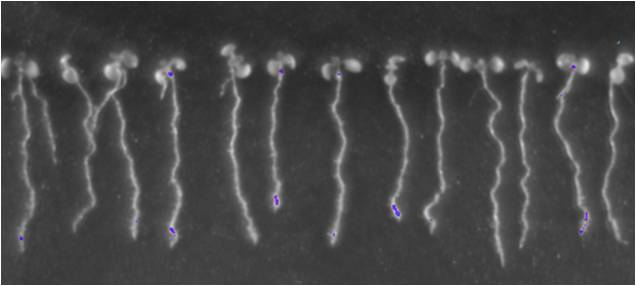


(**A**)


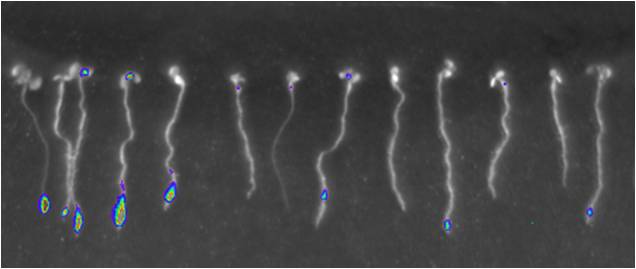


(**B**)

© 2013 by the authors; licensee MDPI, Basel, Switzerland. This article is an open access article distributed under the terms and conditions of the Creative Commons Attribution license (http://creativecommons.org/licenses/by/3.0/).
